# Synergistic Internal Ribosome Entry Site/MicroRNA-Based Approach for Flavivirus Attenuation and Live Vaccine Development

**DOI:** 10.1128/mBio.02326-16

**Published:** 2017-04-18

**Authors:** Konstantin A. Tsetsarkin, Guangping Liu, Evgeniya Volkova, Alexander G. Pletnev

**Affiliations:** a Laboratory of Infectious Diseases, National Institute of Allergy and Infectious Diseases, National Institutes of Health, Bethesda, Maryland, USA; b Center for Biologics Evaluation and Research, U.S. Food and Drug Administration, Silver Spring, Maryland, USA; GSK Vaccines

**Keywords:** attenuation, vaccine, flavivirus, internal ribosome entry site, microRNA

## Abstract

The recent emergence of Zika virus underscores the need for new strategies for a rapid development of safe flavivirus vaccines. Using another flavivirus (Langat virus [LGTV]) that belongs to the group of tick-borne flaviviruses as a model, we describe a dual strategy for virus attenuation which synergistically accesses the specificity of microRNA (miRNA) genome targeting and the effectiveness of internal ribosome entry site (IRES) insertion. To increase the stability and immunogenicity of bicistronic LGTVs, we developed a novel approach in which the capsid (C) protein gene was relocated into the 3′ noncoding region (NCR) and expressed under translational control from an IRES. Engineered bicistronic LGTVs carrying multiple target sequences for brain-specific miRNAs were stable in Vero cells and induced adaptive immunity in mice. Importantly, miRNA-targeted bicistronic LGTVs were not pathogenic for either newborn mice after intracranial inoculation or adult immunocompromised mice (SCID or type I interferon receptor knockout) after intraperitoneal injection. Moreover, bicistronic LGTVs were restricted for replication in tick-derived cells, suggesting an interruption of viral transmission in nature by arthropod vectors. This approach is suitable for reliable attenuation of many flaviviruses and may enable development of live attenuated flavivirus vaccines.

## IMPORTANCE

The recent emergence of Zika virus underscores the need for new strategies for a rapid development of safe flavivirus vaccines. Allied separately attenuating approaches based on (i) microRNA genome targeting and (ii) internal ribosome entry site insertion are not sufficient for relievable attenuation of neurotropic flavivirus pathogenesis. Here, we describe a novel dual strategy that combines the specificity of miRNA-based and the effectiveness of IRES-based attenuating approaches, allowing us to overcome these critical limitations. This developed approach provides a robust platform for reliable attenuation of many flaviviruses and may enable development of live flavivirus vaccines.

## INTRODUCTION

Tick-borne flaviviruses (TBF) constitute a monophyletic single group within the *Flavivirus* genus (family *Flaviviridae*) that harbors causative agents of severe encephalitic (and, less frequently, hemorrhagic) disease in humans (1). Today, inactivated vaccines are available against only the prototype member of the group, tick-borne encephalitis virus (TBEV). Hampered by lengthy vaccination schedules and reliance on additional booster immunizations, the inactivated vaccines appear to be impractical for use in many countries where TBEV is endemic (2, 3). However, despite decades of active research, a single-dose live attenuated vaccine against any member of the TBF group has yet to be developed. This lag in progress is largely due to the absence of an effective strategy for reliable attenuation of TBF pathogenesis.

Recently, we demonstrated that targeting genomes of several TBF, including Langat virus (LGTV) and chimeric TBEV/Langat viruses, using central nervous system (CNS)-specific microRNAs (miRNAs) resulted in a selective attenuation of viral replication in the CNS but not in peripheral organs (4–6). However, considerable instability of the inserted miRNA target sequences in the TBF genome has been observed during viral infection of the CNS, indicating that reliance on only miRNA targeting might not be sufficient for the strong and consistent virus attenuation necessary for a safe vaccine candidate (7, 8). To overcome this pitfall, here we describe a dual strategy combining the specificity of miRNA targeting and the robustness of encephalomyocarditis virus (EMCV) internal ribosome entry site (IRES) insertion for virus attenuation (9, 10). The engineered bicistronic, miRNA-targeted LGTVs, which express C protein under the control of an IRES, were not pathogenic to newborn Swiss Webster (SW), adult immunodeficient SCID, or type I interferon receptor knockout mice, while they induced strong adaptive immunity in normal C3H mice. Moreover, bicistronic LGTVs were restricted for replication in tick-derived cells, thus mitigating issues of their environmental safety (11) as vaccine viruses.

## RESULTS AND DISCUSSION

### Development of a bicistronic miRNA-targeted LGTV.

**FIG 1:**
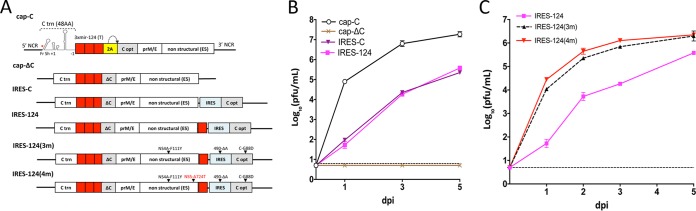
Development of bicistronic LGTV. (A) Schematic representation of the viral genomes constructed for the study. “C trn (48AA)” denotes the replication promoter region of the C gene. The ORF-shifting insertion (red asterisk, nt position 151 of the LGTV genome) of a single A nucleotide (Fr Sh +1) and an ORF restoration (−1) are indicated. Yellow and red boxes denote the 2A protease gene of FMDV and mir-124 target (T) sequences, respectively. Gray boxes denote codon-optimized sequences of the C gene. (B and C) Growth kinetics of recovered viruses in Vero cells. Individual samples for each time point were titrated in Vero cells in duplicate. Results are presented as an average ± standard deviation (shown as error bar). The dashed line indicates the limit of virus detection (0.7 log_10_ PFU/ml).

We modified the E5 strain of LGTV by inserting three target (T) copies of brain-specific mir-124 miRNA into duplicated C gene regions (DCGR) using a strategy previously described (12). The resulting monocistronic virus (cap-C) independently expresses regulatory (C-trn [truncated]) and structural C-opt [codon-optimized]) functions of the C gene, using the 2A protease of foot-and-mouth disease virus (FMDV) for cleavage of full-length C protein from the nascent polypeptide (Fig. 1A). An open reading frame (ORF)-shifting insertion (+1 nucleotide [nt]) and a deletion (−1 nt) were introduced into C-trn to ensure the genetic stability of the cap-C virus by preventing recombination between two C gene sequences (12). The cap-C virus replicated efficiently in Vero cells; however, deletion of the 2A protease of foot-and-mouth disease virus (FMDV) and 89 N-terminal amino acids (aa) of C-opt completely abolished accumulation of the cap-ΔC virus in Vero cells (Fig. 1B). Insertion of a full-length C-opt gene under the control of the IRES into the 3′ noncoding region (NCR) of cap-ΔC restored accumulation of infectious virus (IRES-C) in Vero cells (Fig. 1B). Interestingly, relocation of all structural genes (C/prM/E) from the N terminus of the LGTV ORF into the 3′ NCR under the control of the IRES resulted in IRES-C/prM/E virus, which did not form detectable infectious foci in Vero cells (see Fig. S1 in the supplemental material). To increase the safety and stability of bicistronic LGTV in the CNS, IRES-C was modified by inserting an additional mir-124(T) sequence in the region between the NS5 gene and the 5′ end of the IRES to generate IRES-124. IRES-124 and IRES-C replicated similarly in Vero cells (Fig. 1B), but only IRES-124 failed to cause mortality in newborn Swiss Webster (SW) mice infected intracranially (i.c.) with a dose of 10^3^ PFU/mouse (Fig. S2), although the differences in survival were not statistically significant (*P* = 0.317, log rank test). Nevertheless, IRES-124 was selected for additional evaluations.

10.1128/mBio.02326-16.1 FIG S1  Relocation of structural genes (C/prM/E) into the 3′ NCR under the control of IRES impairs growth of bicistronic LGTV in Vero cells. (A) Schematic representation of the viral genomes used in the study. To generate IRES-C/prM/E, ΔC/prM/E genes were deleted in IRES-C, preserving 34 C-terminal amino acids of E protein (ΔE). prM/E genes were inserted downstream of the C-opt gene of IRES-C. (B) Vero cell monolayers in 12.5-cm^2^ flasks were transfected with 5 µg of IRES-C or IRES-C/prM/E constructs. At 4 dpi, cell culture medium was collected and titrated in Vero cells. The limit of virus detection is 0.7 log_10_ PFU/ml. Download FIG S1, PDF file, 0.1 MB. Copyright © 2017 Tsetsarkin et al. 2017 Tsetsarkin et al. This content is distributed under the terms of the Creative Commons Attribution 4.0 International license.

10.1128/mBio.02326-16.2 FIG S2  Insertion of an additional copy of mir-124(T) sequence between the NS5 gene and the 5′ end of IRES in IRES-C reduces mortality of newborn SW mice after i.c. infection with bicistronic LGTV. (A) Schematic representation of the viral genomes used in the study. (B) Survival of newborn SW mice (*n* = 10) inoculated i.c. with 10^3^ PFU/mouse of rLGTVs. Differences in survival curves were compared using the log rank (Mantel-Cox) test. Download FIG S2, PDF file, 0.2 MB. Copyright © 2017 Tsetsarkin et al. 2017 Tsetsarkin et al. This content is distributed under the terms of the Creative Commons Attribution 4.0 International license.

To improve the growth of bicistronic LGTV in vertebrate cells and identify Vero cell adaptive mutations, IRES-124 was passaged 10 times in Vero cells, followed by genome sequencing. Four mutations were identified, of which three were located outside the prM/E gene region (Table S1). To minimize the effect of cell adaptive mutations on the immunogenicity of LGTV, only mutations located outside the structural prM/E gene region were introduced into the IRES-124 genome. The titer of the resulting IRES-124(3m) virus in Vero cells was increased 50-fold compared to IRES-124 (Fig. 1C). To assess the immunogenicity of bicistronic LGTV, adult C3H mice were inoculated intraperitoneally (i.p.) with a dose of 10^5^ PFU of IRES-124(3m). All animals (*n* = 5) remained free of neurological disease while developing a mean neutralizing antibody of 204 ± 161 (reciprocal 50% plaque reduction neutralization assay [PRNT_50_]) by day 28 (Table S2). Moreover, viremia was not detected in any of the immunized mice on day 1 post-challenge with 10^4^ PFU of wild-type (wt) LGTV, whereas all mock-inoculated mice became viremic (Table S2) with a mean virus titer of 2.7 ± 0.4 log_10_ PFU/ml.

10.1128/mBio.02326-16.6 TABLE S1  Substitutions identified in IRES-124 after 10 passages in Vero cells. Numbers indicate the position of the mutation within the gene or protein. N/A, not applicable. Download TABLE S1, DOCX file, 0.04 MB. Copyright © 2017 Tsetsarkin et al. 2017 Tsetsarkin et al. This content is distributed under the terms of the Creative Commons Attribution 4.0 International license.

10.1128/mBio.02326-16.7 TABLE S2  Immunogenicity and protective efficacy of IRES-124(3m) virus in 3-week-old C3H mice Three-week-old C3H mice (female) were infected intraperitoneally with 10^5^ PFU of IRES-124(3m) or mock inoculated with L-15 medium supplemented with 1× SPG and monitored for neurological symptoms daily until 28 dpi. At 29 dpi, mice were challenged with 10^4^ PFU of LGTV (strain TP-21) and monitored for morbidity for an additional 28 days. Mice were bled at 1 and 30 dpi for detection of virus in the serum and at 28 and 56 dpi for measurement of neutralizing antibody titer (presented as a geometric mean) using the 50% plaque reduction neutralization assay (PRNT_50_) against the LGTV TP-21 strain as described previously (29). ^a^, virus load in the serum was determined by titration in Vero cells. Limit of virus detection was 1.7 log_10_(PFU/ml). Download TABLE S2, DOCX file, 0.1 MB. Copyright © 2017 Tsetsarkin et al. 2017 Tsetsarkin et al. This content is distributed under the terms of the Creative Commons Attribution 4.0 International license.

To assess the stability of IRES-124(3m) in vertebrate cells, we passaged the virus an additional 10 times in Vero cells. Sequence analysis identified accumulation of two novel mutations: one in the E and one in the NS5 gene region (Table S3). The latter (NS5-A724T) substitution was incorporated into IRES-124(3m). The resulting IRES-124(4m) virus replicated slightly more efficiently than IRES-124(3m) (Fig. 1C) and was selected for further evaluation.

10.1128/mBio.02326-16.8 TABLE S3  Substitutions identified in IRES-124(3m) after 10 passages in Vero cells. ^a^, numbers indicate position of the mutation within the gene or protein. Download TABLE S3, DOCX file, 0.03 MB. Copyright © 2017 Tsetsarkin et al. 2017 Tsetsarkin et al. This content is distributed under the terms of the Creative Commons Attribution 4.0 International license.

### Characterization of bicistronic LGTV in animals.

**FIG 2:**
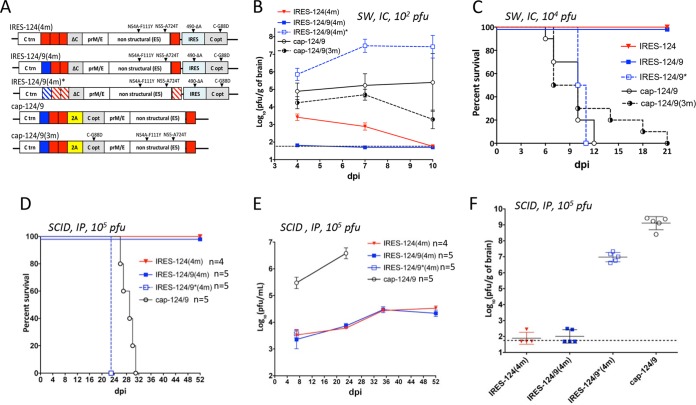
Synergistic effect between IRES- and miRNA-based strategies attenuates neuropathogenesis and neuroinvasiveness of bicistronic LGTVs in mice. (A) Schematic representation of recombinant LGTV (rLGTV) genomes used in this study. Red and blue boxes denote mir-124(T) and mir-9(T) sequences, respectively. Striped boxes indicate scrambled (synonymous) sequence for mir-124(T) and mir-9(T). (B) Growth kinetics of rLGTVs in the brains of newborn Swiss Webster (SW) mice, after i.c. inoculation with 10^2^ PFU. Viral load in the brain (*n* = 3) ± standard deviation (SD) was determined by titration in Vero cells. (C) Survival of newborn SW mice (*n* = 10) inoculated i.c. with 10^4^ PFU/mouse of rLGTVs. (D) Survival of SCID mice (*n* = 4 or 5) inoculated i.p. with 10^5^ PFU/mouse of rLGTVs. (E and F) Mean titer in the serum (E) and brain (F) of SCID mice after i.p. infection with 10^5^ PFU/mouse. (E) Due to the death or paralysis of the animals, mouse serum samples from IRES-124/9(4m)*- and cap-124/9-infected groups were not collected after days 7 and 23, respectively. (F) Brains from healthy mice infected with IRES-124(4m) and IRES-124/9(4m) were collected on day 52. Brains from paralyzed/dead mice were isolated on day 23 [IRES-124/9(4m)* group] or from day 26 to 31 (cap-124/9 group). The dashed line indicates the limit of virus detection (1.7 log_10_ PFU/ml of serum [F] or 1.7 log_10_ PFU/g of brain [F]).

Previously, we showed that miRNA targeting of the LGTV genome with a combination of CNS-specific mir-124 and mir-9 targets is more effective for attenuation of virus neurovirulence than is insertion of only mir-124 targets (5). Therefore, we developed an additional virus in which one of the mir-124(T)s was replaced with mir-9(T) [see IRES-124/9(4m) in Fig. 2A]. To evaluate the effects of miRNA targeting and/or IRES insertion on LGTV growth in the CNS, we infected newborn SW mice in the brain with 100 PFU of either IRES-124/9(4m) or IRES-124(4m) virus. Control mice were infected with IRES-124/9(4m)* virus, which has synonymous mutations in all miRNA(T)s, or with monocistronic cap-124/9 virus, which has the same combination of miRNA(T)s as bicistronic IRES-124/9(4m) (Fig. 2A). Replication of IRES-124/9(4m) and IRES-124(4m) in mouse brain was dramatically reduced compared to that of IRES-124/9(4m)* and cap-124/9 viruses, indicating that both IRES- and miRNA-based approaches contribute to viral attenuation in the CNS (Fig. 2B). The difference in the titers attained by cap-124/9 and IRES-124/9(4m) in the brain was substantially greater than the difference in Vero cells (Fig. 2B and S3), suggesting synergy between IRES- and miRNA-based mechanisms of LGTV attenuation in the CNS. To corroborate these results, we infected newborn SW mice i.c. with a high virus dose (10^4^ PFU) and monitored survival of mice for 21 days. All animals infected with IRES-124/9(4m) and IRES-124(4m) survived infection, whereas all mice infected with IRES-124/9(4m)* or cap-124/9 died by day 12 (Fig. 2C).

10.1128/mBio.02326-16.3 FIG S3  Growth in Vero cells of viruses used in mouse studies. Vero cell monolayers in 12.5-cm^2^ flasks were transfected with 5 µg of plasmid DNA constructs depicted in Fig. 2A. Cell culture medium aliquots collected at indicated time points were titrated in Vero cells in duplicate. Mean virus titers ± SD are shown. The dashed line indicates the limit of virus detection (0.7 log_10_ PFU/ml). Download FIG S3, PDF file, 0.1 MB. Copyright © 2017 Tsetsarkin et al. 2017 Tsetsarkin et al. This content is distributed under the terms of the Creative Commons Attribution 4.0 International license.

To evaluate if the Vero adaptive mutations in bicistronic LGTVs attenuate virus replication in the CNS of mice, we incorporated C-G88D, NS4A-F111Y, and NS5-A724T substitutions from IRES-124/9(4m) into monocistronic cap-124/9, generating cap-124/9(3m) virus (Fig. 2A). Since monocistronic LGTV does not have an IRES, the IRES-490-ΔA deletion was not included in cap-124/9(3m). cap-124/9(3m) replicates less efficiently than cap-124/9 in the brain of newborn SW mice (Fig. 2B) (*P* = 0.006, 2-way analysis of variance [ANOVA]). However, the growth of cap-124/9(3m) was significantly increased over that of IRES-124/9(4m) virus (Fig. 2B) (*P* < 0.001, 2-way ANOVA). In addition, infection of newborn SW mice with cap-124/9(3m) resulted in 100% mortality by 21 days postinfection (dpi) (Fig. 2C). These data demonstrate that although Vero cell adaptive mutations cause moderate LGTV attenuation *in vivo*, they are not sufficient to restrict LGTV pathogenesis.

To evaluate the ability of bicistronic LGTV to invade the CNS from the periphery, 3-week-old SCID mice (*n* = 4 or 5 animals/group) were infected i.p. with 10^5^ PFU of viruses featured in Fig. 2A and monitored for 52 days. SCID mice are severely deficient in the development of functional B and T lymphocytes, permitting prolonged viral replication *in vivo* uninhibited by the development of humoral and cell-mediated adaptive immunity. All mice infected with IRES-124(4m) and IRES-124/9(4m) survived (Fig. 2D) and developed moderate viremia (Fig. 2E) that persisted for the duration of the experiment (52 days). The viral load in the brains at day 52 (Fig. 2F) was approximately 200- to 400-fold lower than the load in the serum. In contrast, 100% of mice infected with cap-124/9 died by day 31 and developed substantial viremia (Fig. 2D and E). Moreover, a high titer of cap-124/9 virus was detected in the brains of paralyzed animals (approximately 100-fold higher than viral load in the serum). Sequence analysis of cap-124/9 recovered from the brains of morbid mice revealed accumulation of deletions in the miRNA(T) region (Fig. S4). Interestingly, bicistronic IRES-124/9(4m)* lacking functional miRNA(T) sequences was also highly neuroinvasive (Fig. 2F), causing paralysis in 100% of SCID mice by day 23, even though viremia produced by this virus was substantially lower than that produced by cap-124/9 on day 7 (Fig. 2E). Together, these data demonstrate that cooperation between IRES- and miRNA-based attenuating mechanisms is instrumental to ensure the safety of bicistronic LGTV in immunocompromised animals. Sequencing analyses of IRES-124(4m) and IRES-124/9(4m) viruses recovered from serum samples collected on day 52 did not identify mutations or deletions in the miRNA(T) region, confirming high genetic stability of bicistronic LGTV *in vivo*.

10.1128/mBio.02326-16.4 FIG S4  Sequence analysis of cap-124/9 viruses recovered from the brain of morbid SCID mice. SCID mice (*n* = 5) were infected i.p. with 10^5^ PFU/mouse. Mice succumbed to encephalitis between 26 and 31 dpi. Brains from paralyzed mice were collected and used for viral isolation and sequencing analysis. Red double arrows schematically indicate the location of deleted sequences in the cap-124/9 genome. Download FIG S4, PDF file, 0.1 MB. Copyright © 2017 Tsetsarkin et al. 2017 Tsetsarkin et al. This content is distributed under the terms of the Creative Commons Attribution 4.0 International license.

The CNS titer of IRES-124/9(4m)* was higher than that of cap-124/9 in newborn SW mice but not in the adult SCID mice infected i.p. (Fig. 2B and F). This observation is consistent with the fact that all brain-isolated cap-124/9 viruses from SCID mice contained deletions in all miRNA target regions (Fig. S4). These escape mutations were selected following at least 27 days of viral replication in a peripheral area prior to the onset of encephalitis. In the absence of an miRNA-mediated mechanism of attenuation, LGTVs with monocistronic genome organization are expected to replicate more efficiently in the CNS than bicistronic viruses. In contrast, in the experiment using newborn SW mice, viruses were directly injected into the CNS, and brain samples were collected before the escape mutations in miRNA target regions of cap-124/9 could be detected.

**FIG 3:**
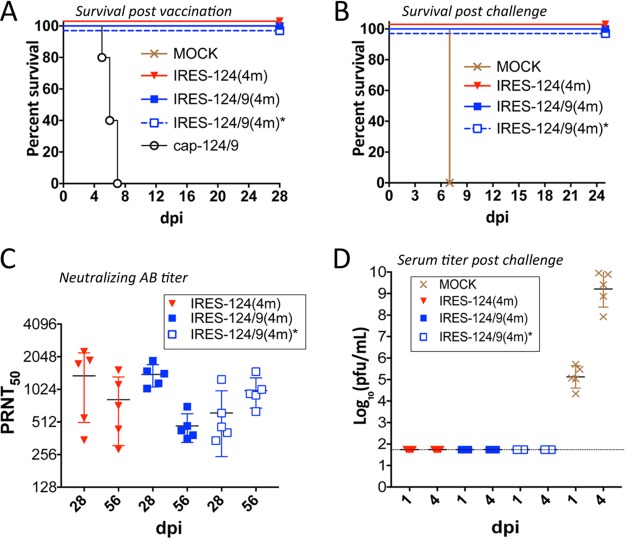
Adult B6 IFNRI^−/−^ mice immunized with bicistronic LGTVs are protected against lethal challenge with wt LGTV. (A) Survival of B6 IFNRI^−/−^ mice (*n* = 5) after immunization. Mice were inoculated i.p. with 10^5^ PFU/mouse of rLGTVs or with diluent alone (mock). (B) Survival of vaccinated mice after i.p. challenge with 10^2^ PFU of wt LGTV. At 31 days postvaccination, animals were challenged with wt LGTV and monitored for neurological signs of disease. (C) Neutralizing antibody titer in the serum of B6 IFNRI^−/−^ mice at 28 and 56 days postinoculation with bicistronic LGTVs. Neutralizing antibody titer was determined using the 50% plaque reduction neutralization assay (PRNT_50_) against wt LGTV. (D) Titer of wt LGTV in the serum of mice on days 1 and 4 postinfection. B6 IFNRI^−/−^ mice immunized with bicistronic LGTVs or mock-infected animals were challenged on day 32 with 10^2^ PFU of wt LGTV. Virus titer in the serum was determined by titration in LLC-MK2 cells. The dashed line indicates the limit of virus detection (1.7 log_10_ PFU/ml of serum).

Immunization of C3H mice with bicistronic LGTV induced an adaptive immune response. However, immunocompetent C3H mice quickly developed resistance to wt LGTV challenge, which limits their use in lethal challenge studies (Table S2). To overcome this limitation, we used adult type I interferon receptor-deficient mice (B6 IFNRI^−/−^), which have been shown to be sensitive to flavivirus infection (13, 14). B6 IFNRI^−/−^ mice were inoculated i.p. with 10^5^ PFU of viruses listed in Fig. 2A or with diluent alone (mock), followed by challenge with 10^2^ PFU of wt LGTV at day 32. All mice infected with bicistronic LGTVs survived the primary inoculation (Fig. 3A) and challenge with wt LGTV (Fig. 3B) and developed LGTV-specific neutralizing antibodies (Fig. 3C). In contrast, all cap-124/9-inoculated mice died by day 7 (Fig. 3A), and all mock-inoculated mice succumbed to wt LGTV challenge by day 7 (Fig. 3B). Sequence analysis of the viruses recovered from the brain and spleen of 2 morbid mice infected with cap-124/9, which died at day 7, showed that all miRNA(T) sequences remained stable. These data suggest that in contrast to 3-day-old SW and adult SCID mice, the IRES-based attenuation approach alone is sufficient to prevent pathogenesis of LGTV in B6 IFNRI^−/−^ mice.

It appears that in the absence of a type I interferon response, the replication of monocistronic LGTV occurs uncontrollably, resulting in multiorgan failure rather than encephalitis. In agreement with this explanation is the fact that dying mice did not exhibit symptoms of neurological disease (encephalitis and paralysis) and that escape mutations in miRNA targets were not detected in cap-124/9 virus isolated from the brain of the infected B6 IFNRI^−/−^ mice. In contrast, the IRES-mediated attenuation mechanism ensures sufficient reduction of LGTV replication in peripheral organs, while induction of adaptive immune response clears the virus, which has potential to invade the CNS [see IRES-124/9(4m)* in Fig. 2D to F], prior to the onset of encephalitis.

Viremia was not detected after the challenge of immunized mice with wt LGTV. In contrast, all mock-infected animals developed high viremia (approximately 9 log_10_ PFU/ml [Fig. 3D]). Challenge with wt LGTV on day 32 did not increase neutralizing antibody titer (Fig. 3C), suggesting that bicistronic LGTVs can potentially be used as a single-dose vaccine candidate against neurotropic tick-borne flaviviruses.

### Bicistronic LGTVs are growth restricted in tick-derived cells.

**FIG 4:**
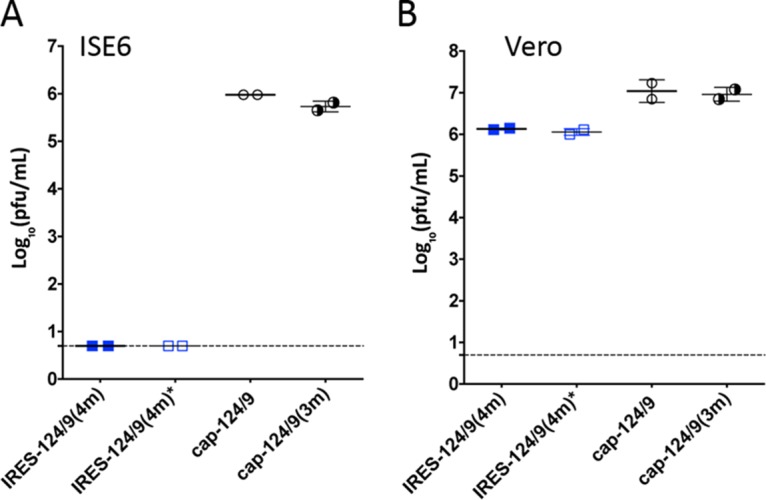
Bicistronic LGTVs are growth restricted in tick-derived cells. Tick-derived ISE6 cells (A) or Vero cells (B) were infected with mono- or bicistronic LGTVs at an MOI of 0.1 in duplicate wells of 6-well plates. Virus titer in cell supernatant was determined on day 5 p.i. Mean virus titers ± SD are shown. The dashed line indicates the limit of virus detection (0.7 log_10_ PFU/ml).

Like all tick-borne flaviviruses, LGTV uses ticks for natural transmission between vertebrate hosts. Since RNA translation from the EMCV IRES is inhibited in cells of insect origin (15), we hypothesized that IRES-based C gene expression in bicistronic LGTVs might also restrict virus growth in tick-derived cells. To validate this hypothesis, we infected ISE6 (derived from *Ixodes scapularis* ticks) or simian Vero cells with bicistronic IRES-124/9(4m) and IRES-124/9(4m)* or monocistronic cap-124/9 and cap-124/9(3m) viruses at a multiplicity of infection (MOI) of 0.1 PFU/cell (Fig. 2A). Both bicistronic viruses with and those without functional miRNA(T)s failed to replicate in tick-derived cells but not in vertebrate Vero cells (Fig. 4). In contrast, cap-124/9 and cap-124/9(3m) replicated efficiently in both cell lines. This result indicates that reliance on IRES for C gene expression (but not Vero cell adaptive mutations) is directly responsible for growth restriction of bicistronic LGTVs in tick-derived cells. We, therefore, concluded that bicistronic genome organization not only attenuates the virulence of the neurotropic flavivirus in the vertebrate host but also can ensure environmental safety of designed viruses by preventing their transmission in nature.

### IRES-dependent gene expression in bicistronic LGTV is strongly attenuated compared to the cap-dependent mechanism.

**FIG 5:**
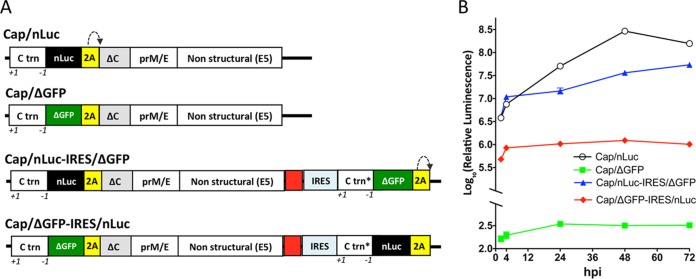
Relative efficiency of IRES-dependent and cap-dependent mechanisms of gene expression in bicistronic LGTV. (A) Schematic organization of subgenomic LGTV replicons expressing nLuc gene via IRES- or cap-dependent mechanisms. The replicons were created by modifying IRES-124(4m) virus; nLuc sequence is indicated as a black box, partial GFP sequence (ΔGFP) is represented by a green box, and 2A protease is shown as a yellow box. The target sequence for mir-124 is shown in red. Frameshift and frame restoration in Ctrn and Ctrn* are represented by +1 and −1, respectively. (B) Relative luminescence kinetics for the LGTV replicons transfected into Vero cells. Luminescence values are calculated as mean ± SD (shown as error bar) from 3 biological replicates of Vero cell extracts transfected with replicon RNAs.

To evaluate the relative expression of LGTV genes in bicistronic viruses, we constructed a panel of subgenomic LGTV replicons, which express the nanoluciferase (nLuc) gene via IRES- or cap-dependent mechanisms (Fig. 5A). For that, the cDNA clone of IRES-124(4m) virus was modified by replacing miRNA(T)s located in the ORF with the sequence of the nLuc gene and by deletion of mir-124(T), IRES, and C-opt sequences in the 3′ NCR. In addition, a cytomegalovirus (CMV) promoter was replaced with an SP6 promoter for *in vitro* RNA transcription followed by deletion of intron sequences in the LGTV ORF. In the resulting Cap/nLuc replicon, the nLuc gene is expressed as a fused protein with C-trn and 2A protease sequences in a cap-dependent manner. Replacement of the nLuc gene in Cap/nLuc with a partial sequence of the green fluorescent protein gene (ΔGFP, nt 259 to 717) prevented accumulation of nLuc signal in the Cap/ΔGFP replicon-transfected Vero cells (Fig. 5B). Insertion of mir-124(T) followed by IRES, C-trn* (which translates into the same protein as C-trn but contains synonymous mutations in every codon), ΔGFP, and 2A protease sequences into the 3′ NCR of Cap/nLuc has no effect on cap-dependent nLuc expression from the Cap/nLuc-IRES/ΔGFP replicon, compared to Cap/nLuc at 2 and 4 h post-RNA transfection. However, modest reduction of nLuc activity (from 3.5- to 8-fold) was detected at the later time points, when active RNA replication is established (Fig. 5B). This correlates with a reduction of relative RNA abundance for the Cap/nLuc-IRES/ΔGFP replicon compared to Cap/nLuc and Cap/ΔGFP (Fig. S5). In contrast, insertion of C-trn*-nLuc-2A sequence under the translation control of IRES (see Cap/ΔGFP-IRES/nLuc in Fig. 5A) strongly reduces nLuc activity at all time points compared to Cap/nLuc-IRES/ΔGFP. However, the relative abundance of Cap/ΔGFP-IRES/nLuc replicon RNA in Vero cells remained comparable to that of Cap/nLuc-IRES/ΔGFP (Fig. S5). Together, these findings demonstrate that at least two mechanisms are involved in the attenuation of bicistronic LGTV replication: (i) general reduction of genomic RNA replication and (ii) selective reduction in the relative C protein expression compared to cap-dependent expression of the remaining genes of LGTV.

10.1128/mBio.02326-16.5 FIG S5  Relative abundance of the LGTV replicons’ RNA in Vero cells at 24 (A) and 48 (B) h posttransfection. Vero cells were transfected with equal amounts of LGTV replicons’ RNA. At 24 and 48 hpi, total RNA was extracted from 3 independent wells of Vero cells (presented as individual points on the graph). Relative RNA abundance of each replicon’s RNA was calculated as a 2 in the power of α, where α was calculated as the difference between *C*_*T*_ values of each sample and the average *C*_*T*_ value of Cap/nLuc-IRES/ΔGFP replicon (shown as the dashed line). Results are presented as mean ± SD (shown as error bars). Download FIG S5, PDF file, 0.1 MB. Copyright © 2017 Tsetsarkin et al. 2017 Tsetsarkin et al. This content is distributed under the terms of the Creative Commons Attribution 4.0 International license.

Attempts had been made earlier to develop bicistronic neurotropic flaviviruses, which utilized a genetic configuration substantially different from the one described here (16, 17). Viruses generated in those studies either were genetically unstable, quickly reverting to wt (monocistronic) configuration (16), or replicated to low titers (17, 18). Importantly, the previously developed approaches did not incorporate a mechanism to selectively restrain virus neuropathogenesis in the CNS and were associated with significant neurovirulence in different mouse models, limiting their application for vaccine development. The combinational approach described here, which employs IRES-based attenuation and miRNA-mediated suppression of encephalitic flavivirus replication, successfully overcomes such limitations, providing a reliable attenuation platform without compromising virus stability and safety. Thus, superior stability of bicistronic LGTVs in cell culture and *in vivo* (in SCID mice) was achieved by separation of previously characterized promoter (regulatory) and structural functions of the C gene (5) and incorporation of codon-optimizing and/or ORF-shifting mutations into both copies of the C gene.

Translation of the C gene under IRES control represents an important improvement in the design of bicistronic flaviviruses compared to approaches utilizing IRES-mediated expression of the prM/E genes (17, 18) or the entire flavivirus polyprotein (16). A replicon study indicates that IRES-dependent translation in bicistronic LGTV is substantially attenuated compared to the cap-dependent gene expression (Fig. 5). This suggests a selective reduction in the C protein abundance (compared to other viral proteins) in the cells infected with bicistronic LGTVs. However, in contrast to prM/E genes, reduction in C gene expression would likely cause little effect on induction of antiviral immunity. In agreement with that is an observation that complete deletion of the C gene in single-round (replication-defective) or DNA flavivirus vaccines did not interfere with their immunogenicity (3, 19–23). We, therefore, speculate that the developed genetic configuration will drive the superior immunogenic properties of bicistronic flavivirus vaccines compared to strategies developed earlier.

As a final note, we believe that the developed approach should not be confined to TBF but that it can also be applied for development of live vaccines against mosquito-borne flaviviruses such as Japanese encephalitis virus, West Nile virus, and Zika virus (ZIKV). The last has recently emerged as the world’s most significant arbovirus (24). Associated with development of severe birth defects in humans, the ongoing ZIKV outbreaks underscore the need for new strategies (exemplified by this study) for a rapid development of safe vaccines.

## MATERIALS AND METHODS

All experimental protocols were approved by the NIH Institutional Biosafety Committee.

### Plasmids for self-propagating viruses.

Sequence and detailed information for all plasmid constructs are available from the authors upon request. The infectious cDNA clone of the attenuated E5 strain of LGTV under the transcriptional control of the polymerase II (Pol II) promoter from cytomegalovirus and its derivative C48-124(2)/9/1-E5 have been described previously (5). To generate cap-C, C48-124(2)/9/1-E5 was modified by replacing a sequence for mir-9(T) with mir-124(T) and deleting mir-1(T) in the duplicated C gene region (DCGR). cap-ΔC was generated by deleting the 2A protease gene of foot-and-mouth disease virus (FMDV) and 89 N-terminal amino acids (aa) of the C-opt gene in cap-C. To generate IRES-C, the region encoding the IRES sequence from pIRESpuro2 plasmid (Clontech) was fused with the C-opt gene using PCR and inserted after the 8th nt of cap-ΔC. IRES-124 was produced by inserting mir-124(T) after the 6th nt located downstream of the UAA stop codon of the NS5 gene of IRES-C. To generate IRES-124(3m), IRES-124 was subsequently modified by introducing substitutions, U→A at nt position 332 of the NS4A gene (resulting in an F_111_→Y change) and G→A at nt position 263 of the C-opt gene (resulting in a G_88_→D change), and by deletion of a single A residue (ΔA) at nt position 490 of the IRES sequence, generating IRES-124(3m). To generate IRES-124(4m), a G→A substitution was introduced into IRES-124(3m) at nt position 2170 of the NS5 gene (resulting in an A_724_→T change). IRES-124(4m) was modified by replacing the 5′-terminal copy of the mir-124(T) sequence with that of mir-9(T), generating IRES-124/9(4m). IRES-124/9(4m)* was constructed by introducing synonymous mutations into each amino acid codon within miRNA(T) sequences located in the ORF of IRES-124/9(4m). mir-124(T) located between NS5 and the IRES was mutated using the identical “scrambled” sequences as both mir-124(T)s located in the ORF. cap-124/9 was constructed by introducing mir-124(T) sequence into the 3′ NCR of C48-124(2)/9/1-E5 (5) at nt position 14 followed by deletion of mir-1(T) sequence in the DCGR. The NS4A-F111Y, C-G88D, and NS5-A724T substitutions were introduced into cap-124/9 to generate cap-124/9(3m). All plasmids were propagated in the MC1061 strain of *Escherichia coli.*

### Plasmids for LGTV replicons.

LGTV replicons, lacking a functional C gene, were constructed based on IRES-124(4m) plasmid. It contains a pACNR1811 vector and two intron sequences (in the NS1 and NS5 genes of LGTV cDNA, respectively) to reduce plasmid toxicity for *E. coli* (5). To generate Cap/nLuc, we replaced the pACNR1811 vector in IRES-124(4m) with that of pBR322 from the p689 cDNA clone of LGTV (25) and placed viral cDNA under the control of the SP6 promoter. Both intron sequences were deleted. In addition, sequence for miRNA(T)s located in the ORF was replaced with the nanoluciferase (nLuc) gene. To ensure the release of nLuc protein from an LGTV polypeptide, the 2A protease gene from FMDV was inserted in frame at the 3′ end of the nLuc gene, generating the Cap/nLuc-X plasmid. The remaining mir-124(T) and IRES-C-opt sequences in the Cap/nLuc-X plasmid were deleted to restore the authentic 3′ NCR of the LGTV. To generate Cap/ΔGFP, the nLuc gene in the Cap/nLuc plasmid was replaced with the 3′-terminal sequence of the green fluorescent protein (GFP) gene (nt 259 to 717 encoding 153 amino acids). To generate Cap/nLuc-IRES/ΔGFP, the C-opt gene in the Cap/nLuc-X plasmid was replaced with the C-trn-ΔGFP-2A protein gene derived from Cap/ΔGFP. Since 2A protease cleaves itself between the 17th and 18th amino acids, the 18th C-terminal codon (Pro) in the 2A protease gene was replaced with the TAA stop codon. To prevent potential influence by *cis*-acting elements in the C-trn sequence located downstream of the IRES on the Cap/nLuc-IRES/ΔGFP replication, C-trn was modified by introducing synonymous mutations in every amino acid codon to generate a C-trn* sequence. To generate Cap/ΔGFP-IRES/nLuc, we replaced a ΔGFP sequence in Cap/nLuc-IRES/ΔGFP with the nLuc gene. In addition, the mir-124(T)-IRES-C-trn*-nLuc-2A sequence from Cap/nLuc-IRES/nLuc was inserted into Cap/ΔGFP plasmid after the 6th nt of the 3′ NCR of LGTV. Plasmids encoding all LGTV replicons were propagated in the DB1528 strain of *E. coli*. All plasmids were purified using the Endo-Free plasmid maxikit (Qiagen, Valencia, CA) according to the manufacturer’s instructions.

### Cells.

Vero (*Cercopithecus aethiops* [African green monkey] kidney) and LLC-MK2 (*Macaca mulatta* kidney) cells were maintained at 37°C and 5% CO_2_ in Opti-Pro medium (Invitrogen) supplemented with 2% fetal bovine serum (FBS) and 50 µg/ml of gentamicin (26). Tick ISE6 cells derived from *Ixodes scapularis* (27) were maintained at 34°C in 66% Leibovitz L-15 medium (Invitrogen) supplemented with 3.3% FBS (Lonza), 6.6% tryptose phosphate broth solution (MP Biomedicals, Irvine, CA), 0.66% bovine cholesterol lipoprotein concentrate (MP Biomedicals), 50 µg/ml of gentamicin (Invitrogen), 0.296 g/liter of l-aspartic acid, 0.333 g/liter of l-glutamine, 0.3 g/liter of l-proline, 0.323 g/liter of l-glutamic acid, 0.296 g/liter of α-ketoglutaric acid, 11.9 g/liter of d-glucose, and 1× mineral and vitamin solution as described earlier (6, 28).

### DNA transfection, virus recovery, and titration.

Viruses were rescued from plasmid DNA using the Lipofectamine 2000 transfection method as described previously (12). Briefly, 5 µg of plasmid DNA diluted in 0.25 ml of Opti-MEM (Invitrogen) was mixed with 10 µg of Lipofectamine 2000 (Invitrogen) diluted in 0.25 ml of Opti-MEM. The 0.5-ml DNA-Lipofectamine 2000 solution was used for transfection of 1.5 × 10^6^ Vero cells seeded onto a 12.5-cm^2^ flask for 4 h at 37°C and 5% CO_2_. Cells were washed twice with 5 ml of Opti-MEM and maintained in 5 ml of Dulbecco modified Eagle medium (DMEM) (Invitrogen) supplemented with 10% FBS and 1× penicillin-streptomycin-glutamine (PSG) solution for 5 days at 37°C and 5% CO_2_. Aliquots of cell culture medium (0.5 ml) were stored at −80°C and titrated in Vero cells using an immunostaining plaque-forming assay described previously (26). Infectious foci in the methanol-fixed Vero cell monolayers were visualized using immunostaining with TBEV-specific antibodies in hyperimmune mouse ascitic fluid and peroxidase-labeled anti-mouse IgG (Dako Co., Carpinteria, CA).

### Serial passaging and genetic stability of bicistronic LGTVs in Vero cells.

Viruses in the supernatant of Vero cells were harvested on day 5 post-DNA transfection and were diluted in Opti-Pro medium to 2 × 10^4^ PFU/ml, and 1 ml was used to infect new Vero cell monolayers in 25-cm^2^ flasks (MOI, ~0.01). Cells were maintained in 5 ml of Opti-Pro medium at 37°C and 5% CO_2_ for 5 days or until cytopathic effect (CPE) was observed (for IRES-124, it occurs between 7 and 10 dpi). The supernatant was harvested and diluted 1/10 with Opti-Pro medium, and 1 ml of inoculum was used to infect fresh Vero cells in 25-cm^2^ flasks. The process was repeated 10 times. At the end of the 10th passage, viral RNA was extracted from 0.14 ml of supernatant using the QIAamp viral RNA minikit (Qiagen) and PCR amplified using the Transcriptor one-step reverse transcription-PCR (RT-PCR) kit (Roche), followed by sequence analysis of the complete viral genome.

### Replicon study.

Plasmid DNAs (5 µg) of LGTV replicons were linearized with EcoRV restriction endonuclease (New England Biolabs, Ipswich, MA), and 5′-capped RNA transcripts were generated by *in vitro* transcription from the SP6 promoter using the mMessage mMachine kit (Ambion, Austin, TX). Five micrograms of RNA transcripts was electroporated into 5 × 10^7^ Vero cells using a Gene Pulser XCell electroporation system (Bio-Rad, Hercules, CA) as described previously (12). Cells were diluted in 50 ml of Opti-Pro medium supplemented with 2% FBS, 2 mM l-glutamine, and 5 µg/ml of gentamicin. One milliliter of cell suspension was plated into one well of a 24-well plate. Following 2 h of incubation at 37°C, medium in the well was replaced with 1 ml of fresh Opti-Pro medium to remove unattached and dead cells. Cells were incubated at 37°C and 5% CO_2_ until nLuc activity was measured (5) or RNA analysis was performed. For nLuc measurements, cells were washed once with 1 ml of phosphate-buffered saline (PBS) and lysed in 0.3 ml of 1× Pierce luciferase cell lysis buffer (Thermo Scientific) for 10 min at 37°C at constant shaking. Lysates were 10-fold serially diluted in PBS, and 50 µl of sample aliquots was mixed with 50 µl of 2× nanoGlo substrate (Promega). nLuc activity was measured using a Synergy HT microplate reader (BioTek, Winooski, VT). Results are presented as an average from two luminescence value reads for extracts from 3 wells of Vero cells. For RNA analysis, 24 or 48 h posttransfection, cells were washed once with 1 ml of PBS followed by total RNA extraction using 0.75 ml of TRIzol LS reagent (Invitrogen). For each replicon, RNA (0.6 μg) extracted from 3 independent wells of Vero cells was reverse transcribed using the SuperScript III First Strand kit (Invitrogen) according to the manufacturer’s instructions. For each cDNA sample, the threshold cycle (*C*_*T*_) values were determined in duplicate using iQ SYBR green SuperMix (Bio-Rad) with LGTV-specific primers designed to produce an amplicon of 90 bp: LV-qPCR-F (5′-CAAAGGTGGCTGCCAGATG) and LV-qPCR-R (5′-CGCTCTGATCTCTCTTGCAC). Reactions were performed using an ABI 7300 real-time PCR system under the following conditions: 95°C for 10 min followed by 40 cycles of 95°C for 15 s and 60°C for 1 min. Relative RNA abundance was calculated as 2 in the power of α, where α is calculated as the difference between the *C*_*T*_ of each sample and the average *C*_*T*_ value of the Cap/nLuc-IRES/ΔGFP replicon.

### Mouse study.

All mice were purchased from Taconic Inc. All animal study protocols were approved by the NIAID/NIH Institutional Animal Care and Use Committee. All animal experiments were performed in compliance with the guidelines of the NIAID/NIH Institutional Animal Care and Use Committee. The NIAID DIR Animal Care and Use Program acknowledges and accepts responsibility for the care and use of animals involved in activities covered by the NIH IRP’s PHS assurance no. A4149-1, last issued 11 June 2011.

### (i) Replication kinetics of LGTVs in the brains of newborn Swiss Webster mice.

Litters of 10 3-day-old SW mice (Taconic, Hudson, NY) were inoculated intracranially (i.c.) with 100 PFU of virus in 10 µl of L-15 medium supplemented with 1× SPG (218 mM sucrose, 6 mM l-glutamic acid, 3.8 mM KH_2_PO_4_, 7.2 mM K_2_HPO_4_, pH 7.2) as described previously (5). Three pups from each litter were sacrificed at 4, 7, and 10 days postinfection (dpi), and brains were dissected and used to prepare 10% homogenate in L-15–1× SPG solution. Virus titers in the brain suspensions were assayed by titration in Vero cells. The results from this experiment are shown in Fig. 2B.

### (ii) Survival study of newborn SW mice after i.c. infection.

Three 3-day-old SW mice were inoculated i.c. with 10^4^ PFU of virus (corresponding to 3.1 × 10^3^ 50% lethal doses [LD_50_] for the E5 strain of LGTV) diluted in 10 µl of L-15–1× SPG solution. Mice were returned to cages and to their mothers and monitored daily for onset of neurologic symptoms (paralysis) during 21 days, at which point surviving animals were humanely euthanized. The results from this experiment are shown in Fig. 2C.

### (iii) Neuroinvasiveness study in adult immunodeficient SCID mice.

Recombinant LGTVs (rLGTVs) were diluted in L-15–1× SPG solution to a concentration of 10^6^ PFU/ml. Three-week-old male SCID mice (Taconic) in groups of five animals were injected i.p. with 0.1 ml of each virus (dose of 10^5^ PFU/mouse). Mice were monitored for onset of neurologic disease (paralysis) for 52 dpi. Mice were bled at 7, 23, 35, and 52 dpi to assess virus titer in the serum. Brains from mice who survived the experiment (52 dpi) and those who died or developed paralysis after viral infection (23 to 31 dpi) were extracted and homogenized in L-15–1× SPG solution, and virus titer in each homogenate was assessed in Vero cells. Results from this experiment are shown in Fig. 2D to F.

### (iv) Safety and protective efficacy of bicistronic LGTVs in adult B6 IFNRI^−/−^ mice.

rLGTVs (Fig. 2A) were diluted in L-15–1× SPG solution to a concentration of 10^6^ PFU/ml. Three-week-old male B6 IFNRI^−/−^ mice (Taconic) in groups of 5 animals were immunized i.p. with 0.1 ml of each virus (dose of 10^5^ PFU per mouse) or with 0.1 ml of L-15–1× SPG (mock). Mice were monitored for morbidity/mortality for 28 dpi, at which point surviving animals were bled to determine neutralizing antibody titer. At 31 dpi, mice were infected i.p. with 10^2^ PFU of wt LGTV. Mice were bled at 1 and 4 days postchallenge (dpc) to determine the titer of wt LGTV in the serum. At 25 dpc, mice that survived the challenge were bled to determine neutralizing antibody titer using the 50% plaque reduction neutralization assay (PRNT_50_) against the LGTV TP-21 strain as described previously (29). Results from this experiment are shown in Fig. 3.

### (v) Safety and protective efficacy of bicistronic LGTVs in adult C3H mice.

Three-week-old C3H female mice (Taconic) were infected intraperitoneally with 10^5^ PFU of IRES-124(3m) or mock inoculated with L-15 medium supplemented with 1× SPG and monitored daily for signs of neurotropic disease until 28 dpi. At 29 dpi, mice were challenged with 10^4^ PFU of wt LGTV and monitored for morbidity for an additional 28 days. Mice were bled at 1 and 30 dpi (30 dpi corresponds to 1 dpc with wt LGTV) for the detection of virus in serum and at 28 or 56 dpi for measurement of neutralizing antibody against the LGTV TP-21 strain. Results from this experiment are shown in Table S2 in the supplemental material.
