# Supplementary material for: Synergistic Internal Ribosome Entry Site/MicroRNA-Based Approach for Flavivirus Attenuation and Live Vaccine Development
Source: mBio. 2017 Apr 18;8(2):e02326-16. doi: 10.1128/mBio.02326-16 (PMC5395672; doi:10.1128/mBio.02326-16)
Supplement: FIG S1 [file mbo002173275sf1.pdf]

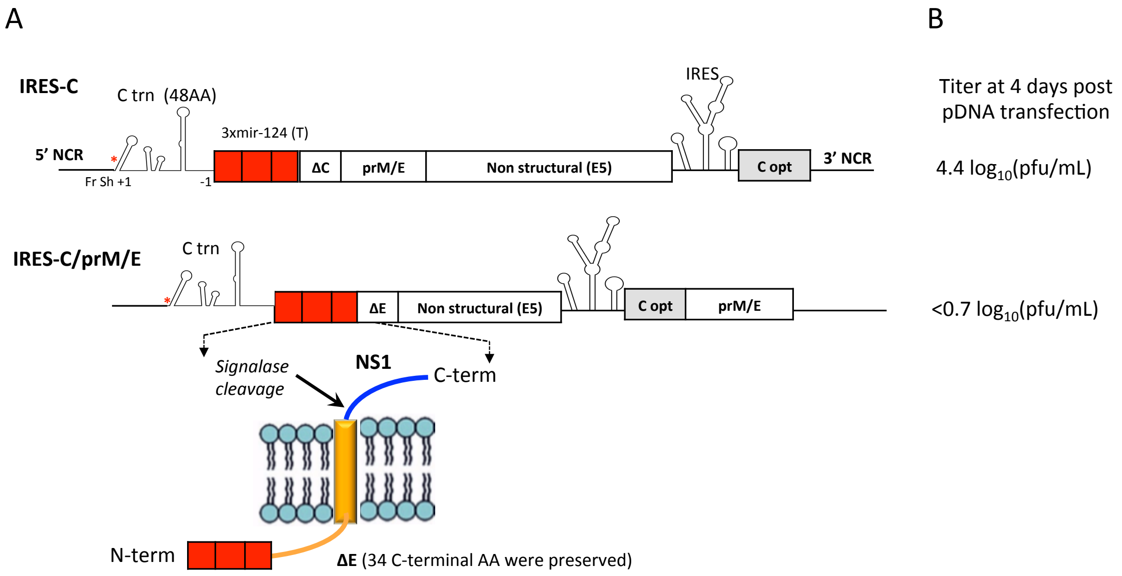

Supplementary Figure S1. Relocation of structural genes (C/prM/E) into the 3'NCR under control of IRES impairs growth of bicistronic LGTV in Vero cells.

(**A**) Schematic representation of the viral genomes used in the study. To generate IRES-C/prM/E, ΔC/prM/E genes were deleted in IRES-C preserving 34 C-terminal AA of E protein (ΔE). prM/E genes were inserted downstream of C-opt gene of IRES-C. (**B**) Vero cell monolayers in 12.5 cm^2 flasks were transfected with 5 µg of IRES-C or IRES-C/prM/E constructs. At 4 dpi, cell culture medium was collected and titrated in Vero cells. Limit of virus detection is 0.7 $\log_{10}$ pfu/mL.
