# Supplementary material for: Synergistic Internal Ribosome Entry Site/MicroRNA-Based Approach for Flavivirus Attenuation and Live Vaccine Development
Source: mBio. 2017 Apr 18;8(2):e02326-16. doi: 10.1128/mBio.02326-16 (PMC5395672; doi:10.1128/mBio.02326-16)
Supplement: FIG S2 [file mbo002173275sf2.pdf]

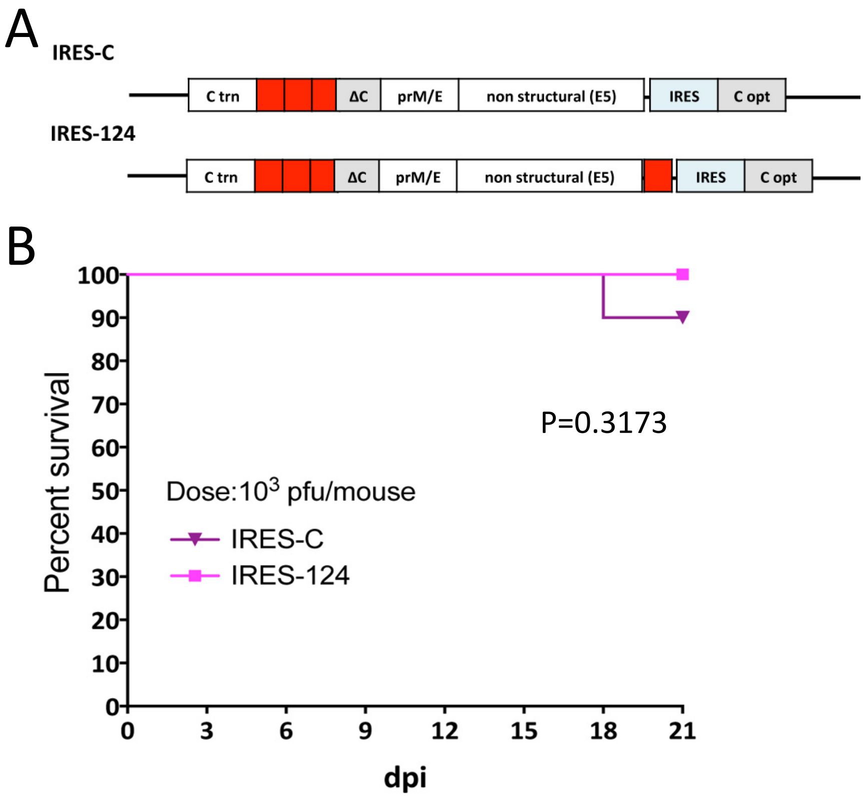


**Supplementary Figure S2. Insertion of an additional copy of mir-124(T) sequence between NS5 gene and 5' end of IRES in IRES-C reduces mortality of newborn SW mice after IC infection with bicistronic LGTV.**

(**A**) Schematic representation of the viral genomes used in the study. (**B**) Survival of newborn SW mice (n=10) inoculated IC with $10^3$ pfu/mouse of rLGTVs. Differences in survival curves were compared using log-rank (Mantel-Cox) test.
