# Supplementary material for: Synergistic Internal Ribosome Entry Site/MicroRNA-Based Approach for Flavivirus Attenuation and Live Vaccine Development
Source: mBio. 2017 Apr 18;8(2):e02326-16. doi: 10.1128/mBio.02326-16 (PMC5395672; doi:10.1128/mBio.02326-16)
Supplement: TABLE S1 [file mbo002173275st1.docx]

**Supplementary Table S1**. Substitutions identified in IRES-124 after 10 passages in Vero cells

| Gene | Nucleotide | Amino acid |
| --- | --- | --- |
| M | G_217_🡪A | A_73_🡪T |
| NS4A | U_332_🡪A | F_111_🡪Y |
| IRES | ΔA_490_ (deletion) | N/A |
| C optimized | G_263_🡪A | G_88_🡪D |

Numbers indicate position of the mutation within the gene or protein.

N/A - not applicable
