# Supplementary material for: Synergistic Internal Ribosome Entry Site/MicroRNA-Based Approach for Flavivirus Attenuation and Live Vaccine Development
Source: mBio. 2017 Apr 18;8(2):e02326-16. doi: 10.1128/mBio.02326-16 (PMC5395672; doi:10.1128/mBio.02326-16)
Supplement: TABLE S2 [file mbo002173275st2.docx]

**Supplementary Table S2.** Immunogenicity and protective efficacy of IRES-124(3m) virus in 3-week old C3H mice

Three-week-old C3H mice (female) were infected intraperitoneally with 10^5^ pfu of IRES-124(3m) or mock-inoculated with L-15 medium supplemented with 1x SPG and monitored for neurological symptoms daily until 28 dpi. At 29 dpi mice were challenged with 10^4^ pfu of LGTV (strain TP-21) and monitored for morbidity for an additional 28 days. Mice were bled on 1 and 30 dpi for detection of virus in the serum and on 28 and 56 dpi for measurement of neutralizing antibody titer (presented as a geometrical mean) using the 50% plaque reduction neutralization assay (PRNT_50_) against LGTV TP-21 strain as described previously (1).

| Virus | No. of mice | Viremia ^a^ at 1 dpi | PRNT_50%_ against LGTV at 28 dpi | Viremia ^a^ at 30 dpi (1 day post challenge) | Survival at 56 dpi | PRNT_50%_ against LGTV at 56 dpi |
| --- | --- | --- | --- | --- | --- | --- |
| mock | 5 | - | - | 2.7±0.4 | 5/5 (100%) | 28.7 ±8.5 |
| IRES-124(3m) | 5 | 2.58±0.19 | 162±158 | <1.7 | 5/5 (100%) | 144±122 |

^a^ – Virus load in the serum was determined by titration in Vero cells. Limit of virus detection was 1.7 Log_10_(pfu/mL).

1. **Pletnev AG, Bray M, Hanley KA, Speicher J, Elkins R.** 2001. Tick-borne Langat/mosquito-borne dengue flavivirus chimera, a candidate live attenuated vaccine for protection against disease caused by members of the tick-borne encephalitis virus complex: evaluation in rhesus monkeys and in mosquitoes. J Virol **75:**8259-8267.
