# Supplementary material for: Synergistic Internal Ribosome Entry Site/MicroRNA-Based Approach for Flavivirus Attenuation and Live Vaccine Development
Source: mBio. 2017 Apr 18;8(2):e02326-16. doi: 10.1128/mBio.02326-16 (PMC5395672; doi:10.1128/mBio.02326-16)
Supplement: TABLE S3 [file mbo002173275st3.docx]

**Supplementary Table S3**. Substitutions identified in IRES-124(3m) after 10 passages in Vero cells

| Gene | Nucleotide ^a^ | Amino acid |
| --- | --- | --- |
| E | G_607_ 🡪A | D_203_🡪N |
| NS5 | G_2170_🡪A | A_724_🡪T |

^a^ - numbers indicate position of the mutation within the gene or protein..
