# Supplementary material for: Synergistic Internal Ribosome Entry Site/MicroRNA-Based Approach for Flavivirus Attenuation and Live Vaccine Development
Source: mBio. 2017 Apr 18;8(2):e02326-16. doi: 10.1128/mBio.02326-16 (PMC5395672; doi:10.1128/mBio.02326-16)
Supplement: FIG S3 [file mbo002173275sf3.pdf]

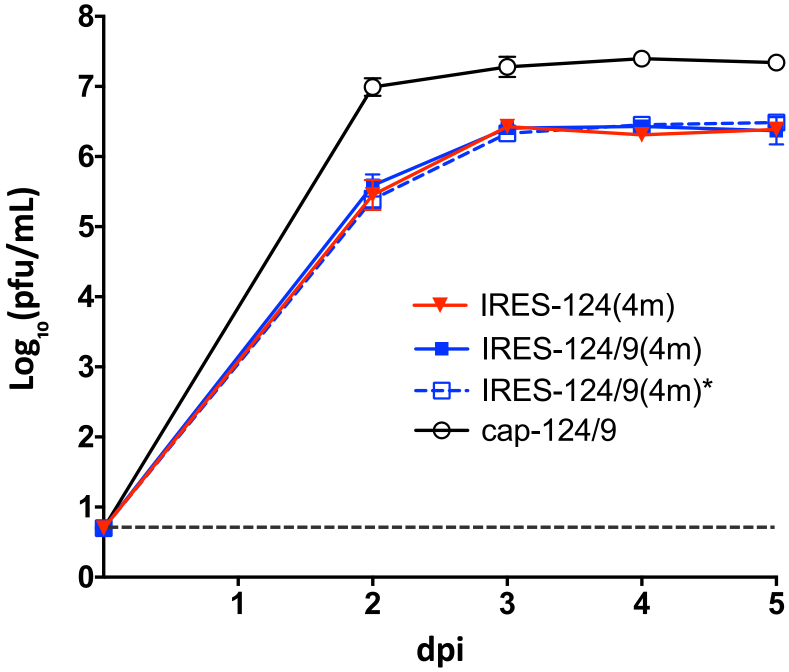

Supplementary Figure S3. Growth in Vero cells of viruses used in mouse studies. Vero cell monolayers in 12.5 cm^2 flasks were transfected with 5 µg of plasmid DNA constructs depicted in **Fig. 2A**. Cell culture medium aliquots collected at indicated time points were titrated in Vero cells in duplicate. Mean virus titers ± SD are shown. The dashed line indicates the limit of virus detection (0.7 log_{10} pfu/mL).
