# Supplementary material for: Synergistic Internal Ribosome Entry Site/MicroRNA-Based Approach for Flavivirus Attenuation and Live Vaccine Development
Source: mBio. 2017 Apr 18;8(2):e02326-16. doi: 10.1128/mBio.02326-16 (PMC5395672; doi:10.1128/mBio.02326-16)
Supplement: FIG S4 [file mbo002173275sf4.pdf]

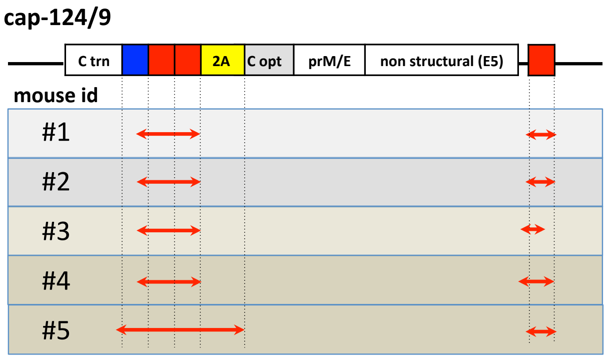

Supplementary Figure S4. Sequence analysis of cap-124/9 viruses recovered from the brain of morbid SCID mice.

SCID mice (n = 5) were infected IP with 10^5 pfu/mouse. Mice succumbed to encephalitis between dpi 26-31. Brains from paralyzed mice were collected and used for viral isolation and sequencing analysis. Red double arrows schematically indicate the location of deleted sequences in the cap-124/9 genome.
