# Supplementary material for: Synergistic Internal Ribosome Entry Site/MicroRNA-Based Approach for Flavivirus Attenuation and Live Vaccine Development
Source: mBio. 2017 Apr 18;8(2):e02326-16. doi: 10.1128/mBio.02326-16 (PMC5395672; doi:10.1128/mBio.02326-16)
Supplement: FIG S5 [file mbo002173275sf5.pdf]

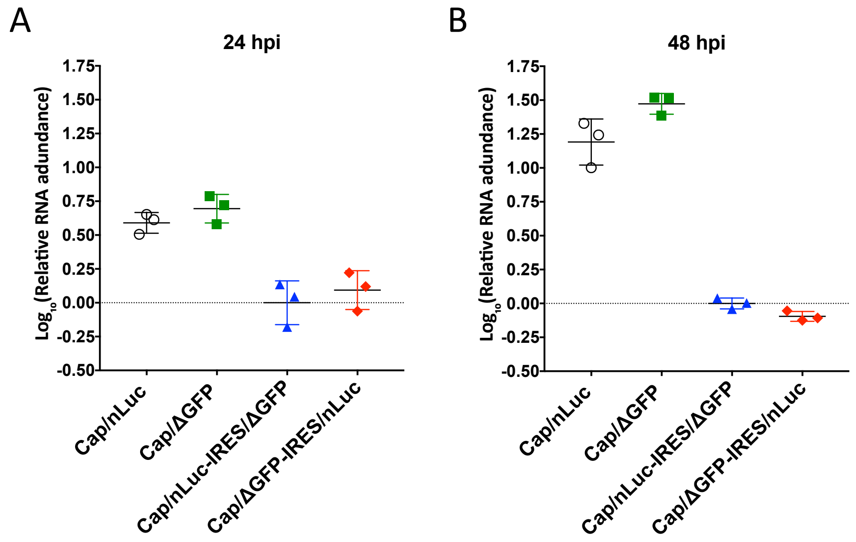

Supplementary Figure S5. Relative abundance of the LGTV replicons' RNA in Vero cells at 24 (A) and 48 (B) hrs post transfection.

Vero cells were transfected with equal amount of LGTV replicons' RNA. At 24 and 48 hpi total RNA was extracted from 3 independent wells of Vero cells (presented as individual points on the graph). Relative RNA abundance of each replicons' RNA was calculated as a 2 in the power of α, where α was calculated as the difference between Ct values of each sample and the average Ct value of Cap/nLuc-IRES/ΔGFP replicon (shown as the dashed line). Results are presented as mean ± SD (shown as error bars).
